# Optimization of Proanthocyanidin Extraction from Grape Seeds Using Response Surface Methodology and Subsequent Evaluation of Its Antioxidant and Immunomodulatory Capacities

**DOI:** 10.3390/foods15071214

**Published:** 2026-04-02

**Authors:** Jiawei Zhang, Yali Yao, Yingjun Ru, Defu Tang

**Affiliations:** 1 College of Animal Science and Technology, Gansu Agricultural University, Lanzhou 730070, China; 17899312684@163.com (J.Z.); 1849166373@163.com (Y.Y.); 2 Diasham Resources Pte. Ltd., Singapore 629314, Singapore; yj.ru@163.com

**Keywords:** UPLC-MS/MS, proanthocyanidins, response surface methodology (RSM), oxidative stress, immunosuppression

## Abstract

This study employed UPLC-MS/MS to determine the contents of major polyphenolic compounds and proanthocyanidins (PCs) in Kyoho grape seeds, optimized the extraction method and conditions for PCs using response surface methodology (RSM), and further evaluated the scavenging activities of PCs against 2,2-diphenyl-1-picrylhydrazyl (DPPH) and hydroxyl (•OH) radicals as well as their effects on growth, immunity, and oxidative stress in mice. Three hundred and sixty 3-week-old male mice (42.28 ± 0.31 g) were assigned to a single factor complete randomized trial design and fed with six different diets including 0 mg/kg vitamin E(VE) + 0 mg/kg PCs, 100 mg/kg VE, 25 mg/kg PCs + 75 mg/kg VE, 50 mg/kg PCs + 50 mg/kg VE, 75 mg/kg PCs + 25 mg/kg VE and 100 mg/kg PCs, respectively. The results demonstrated that PCs were identified as the predominant phenolic compounds, accounting for 29.6% of total phenolic substances in Kyoho grape seeds. Additionally, the ultrasound-assisted extraction method was superior to the shaker-assisted and low-temperature infiltration extraction methods, with optimal conditions of 60% ethanol concentration, material-to-liquid ratio of 1:20 g/mL, temperature of 30 °C, and extraction time of 50 min. Scanning electron microscopy (SEM) revealed that ultrasound treatment effectively disrupted the seed surface structure, facilitating PC release. In vitro, PCs exhibited significantly stronger DPPH and hydroxyl radical (•OH) scavenging activities than vitamin C (VC), Trolox, and gallic acid. Compared with the control group, mice fed diets containing PCs and VE showed higher superoxide dismutase (SOD) activity, glutathione peroxidase (GSH-PX) activity, and total antioxidant capacity (TAOC), Catalase (CAT), GPX and inflammation factor 10 (IL-10) genes levels in the serum and liver (*p* < 0.05), whereas the levels of immunoglobulin G (IgG), immunoglobulin A (IgA), immunoglobulin M (IgM), tumor necrosis factor α (TNF-α), interleukin-1β (IL-1β), and interleukin-6 (IL-6), as well as the mRNA expression of IL-1β and TNF-α, showed the opposite trend (*p* < 0.05). In conclusion, the antioxidant capacity of PCs was stronger than that of VC and VE. The addition of PCs improved the antioxidant activity and immune function of mice.

## 1. Introduction

The grape is one of the most widely consumed fruits globally. The major grape-producing countries are the United States, China, and Europe. In China, grapes, grape leaves, and grape juice have been used in traditional remedies for centuries. Based on their intended use, grapes are classified into wine grapes, table grapes, seedless grapes, seeded grapes, and raisins. Grape seeds are obtained as a by-product of the wine industry. Seeds of red wine grapes contain proanthocyanidins (PCs) [1]. PCs exhibit anti-inflammatory, anti-apoptotic, anti-necrotic, cardioprotective, and anti-cancer activities, and are beneficial for various conditions including skin aging [2]. They also have positive effects on wound healing. PCs demonstrate potent oxygen radical scavenging activity, protecting cells from oxidative stress damage [3]. Numerous studies indicate that PCs can enhance antioxidant defense, for instance, by preventing ischemia/reperfusion injury and carbon tetrachloride-induced liver injury [4,5], alleviating arsenic-induced reproductive oxidative toxicity, and providing protection against cisplatin-induced nephrotoxicity [6]. The antioxidant capacity of PCs has been extensively studied; these compounds can inhibit lipid oxidation and scavenge reactive oxygen species.

PCs have been reported to offer stronger protection against free radical-induced lipid peroxidation and DNA damage compared to VE, VC, and β-carotene [7]. Therefore, this experiment aimed to investigate the antioxidant and immunomodulatory effects of PCs and VE on mouse serum and liver by supplementing mouse diets with varying ratios of PCs and VE, providing a theoretical basis for the potential future use of PCs as a novel feed additive to replace VE. Furthermore, PCs are easy to extract, moderately priced, and exhibit low toxicity [8]. However, the extraction of PCs from grape seeds is often inefficient due to various influencing factors, necessitating the development of specific extraction methods and conditions. Factors such as extraction method, temperature, solvent, solid-to-liquid ratio, and time all affect the PC yield from grape seeds [9]. Several methods have been reported for extracting PCs from grape seeds, including ultrasound, microwave, enzyme-assisted, and mechanically assisted single-solvent or multi-solvent extraction, as well as liquid/liquid phase separation [10,11,12,13]. Additionally, ultrasound has been shown to improve extraction at lower temperatures [14]. Ethyl acetate, acetone, hexane, methanol, ethanol, and water are the most commonly used solvents for PC extraction [15,16]. However, ethanol is more suitable for extracting bioactive compounds than other solvents due to its low toxicity [17]. Therefore, this experiment used ethanol as the extraction solvent and compared ultrasonic-assisted, shaker-assisted, and low-temperature infiltration methods. Response Surface Methodology was employed to screen the optimal extraction method and experimental conditions [18].

The objective of this study was to determine the major components of polyphenols in Kyoho grape seeds, identify the most suitable method for PCs extraction, optimize the extraction conditions, and select the optimal parameters. Furthermore, the antioxidant and immunological effects of dietary supplementation with different proportions of PCs and VE on serum and liver in mice were compared, and the antioxidant capacity and immune function of PCs were evaluated.

## 2. Materials and Methods

### 2.1. Materials

Kyoho grape seeds were obtained from Shiyanghe Winery in Minqin County, Gansu Province. They were air-dried naturally, ground, sieved through a 60-mesh screen, and the resulting powder was defatted using petroleum ether prior to extraction.

### 2.2. Chemical Analysis of Grape Seeds

Grape seed powder (50 mg) was lyophilized and extracted with 1200 μL of pre-cooled (−20 °C) 70% aqueous methanol containing internal standards. The mixture was vortexed intermittently over 3 h, centrifuged at 12,000 rpm for 3 min, and the supernatant was filtered through a 0.22 μm membrane prior to UPLC-MS/MS analysis.

### 2.3. Proanthocyanidin Extraction

One gram of defatted grape seed powder was weighed, and PCs were extracted using ultrasonic-assisted, shaker-assisted, and low-temperature infiltration methods, respectively. Various levels of ethanol concentration, temperature, time, and other parameters were tested throughout the experiment. The extracted samples were centrifuged at 4 °C and 9500× *g* for 10 min. The supernatant was purified via solid-phase extraction to obtain the test solution.

### 2.4. Standard Solution Preparation and Measurement

A 10.0 mg PC standard was dissolved in 10 mL methanol. Aliquots of 0, 0.1, 0.25, 1.0, and 1.5 mL of this solution were transferred to 10 mL volumetric flasks, which were then filled to volume with methanol and mixed. One mL from each was taken for measurement. The measurement method was identical to that used for sample analysis. The PC standard curve was: y = 0.00477x − 0.00327, R^2^ = 0.999.

### 2.5. Sample Measurement

A mixture of n-butanol and hydrochloric acid (95:5, *v*/*v*) was prepared. 0.6 mL of this mixture was placed in a stoppered conical flask, followed by the addition of 20 μL of a 2% ammonium ferric sulfate solution (prepared in 2 mol/L HCl) and 0.1 mL of the test solution. After thorough mixing, the flask was placed in a boiling water bath for reflux and heated precisely for 40 min, then cooled in an ice-water bath. Absorbance was measured at 546 nm. The PC content in the sample was calculated based on the standard curve, and colorimetric measurements were completed within one hour.

The PC content in the sample was calculated using the following formula:

$$X(\%)=\frac{m_{1}\times V\times 1000}{m\times 1000\times 1000}\times 100$$

X = Percentage of PC content in the sample, g/100g

m_1_ = Amount of proanthocyanidins in the reaction mixture, μL

V = Total volume of the sample after preparation, mL

m = Sample mass, mg

### 2.6. Single-Factor Experiments

To screen the optimal method for PCs extraction, the effects of ethanol concentration, solid-to-liquid ratio, time, rotational speed (for shaker), and temperature on PCs yield from grape seeds were evaluated based on PCs content. The tested conditions included: solid-to-liquid ratios of 1:10, 1:20, 1:30, 1:40, 1:50 g/mL; temperatures of 10, 20, 30, 40, 50, 60 °C; ethanol concentrations of 40%, 50%, 60%, 70%, 80%, 90%, 100%; and rotational speeds of 75, 95, 115, 125, 135, 145, 155 rpm. Extraction times for ultrasonic and shaker-assisted methods were 10, 20, 30, 40, 50, and 60 min. For the low-temperature infiltration method, extraction times were 12, 24, 36, 48, 60, and 72 h. All extractions were performed at 4 °C in darkness to minimize oxidative degradation of PCs. When not being assessed, all extraction variables were held constant at: solid-to-liquid ratio 1:30 g/mL, temperature 30 °C (4 °C for low-temperature infiltration), ethanol concentration 60%, rotational speed 115 rpm, and time 30 min (1 h for low-temperature infiltration).

### 2.7. Response Surface Design

RSM was applied to develop a protocol for PC extraction from grape seeds to determine the optimal combination of variables for maximum yield. RSM was used to analyze the effects of different extraction process variables (solid-to-liquid ratio, ethanol concentration, temperature, time, and rotational speed) on PC content. Factor levels were selected based on preliminary tests. The response variable for modeling corresponded to the amount of PCs acquired in each experiment. Design-Expert software (11.0) was used to derive regression equations and perform analysis of variance (ANOVA). ANOVA summarized the results obtained under all experimental conditions. A 95% confidence interval was set to test the significance of factors and their interactions. The F-statistic test was used to assess the adequacy of the regression model in describing the observed data. The R-squared statistic was used to analyze the percentage of variance explained by the optimized parameters. Additionally, normal probability plots of residuals and plots of residuals versus predicted responses were used to evaluate model suitability.

The full design comprised 29 experimental runs (17 for the low-temperature infiltration design), including five replicates of the center point to estimate experimental error. All runs were randomized to minimize the impact of unexpected variability. The experimental factor levels are shown in Table 1.

### 2.8. DPPH and •OH Radical Scavenging Assay

The DPPH assay was performed as previously described [19]. Briefly, 1.0 mL of sample solution at different concentrations (5, 10, 20, 40, 60, 80, and 100 μg/mL) was mixed with 0.50 mL of a 0.4 mmol/L DPPH ethanol solution and 2.0 mL of water. The mixture was vortexed, allowed to react in the dark at 30 °C for 30 min, and the absorbance was measured at 517 nm. VC served as the positive control, and a 90 μm DPPH solution was used as the blank. The DPPH radical scavenging rate for each sample was calculated as:

Scavenging rate (%) = 100 × (A_blank_ − A_sample_)/A_blank_

where A_blank_ is the absorbance of the DPPH solution and A_sample_ is the absorbance of the extract solution. The extract concentration providing 50% scavenging (IC_50_) was calculated from the plotted inhibition percentage versus extract concentration.

For quantitative comparison with reference standards, Trolox and gallic acid were also used as positive controls in the DPPH assay. Standard stock solutions of Trolox (0–200 μmol/L) and gallic acid (0–100 μg/mL) were prepared, and their DPPH scavenging activities were measured under the same conditions. The antioxidant activity was expressed as μmol Trolox equivalents per gram of extract (μmol TE/g).

The hydroxyl radical scavenging capacity was measured using a commercial assay kit (Beyotime Biotechnology, Shanghai, China).

### 2.9. Scanning Electron Microscopy (SEM) Analysis

To visualize the morphological changes in grape seed powder before and after ultrasound-assisted extraction, scanning electron microscopy was performed. Samples were dried at 45 °C for 24 h, mounted on aluminum stubs using double-sided carbon tape, and sputter-coated with gold-palladium to enhance conductivity. Observations were conducted using a scanning electron microscope (Hitachi S-4800, Tokyo, Japan) at an accelerating voltage of 10.0 kV. Images were captured at magnifications of 420× and 20,000× to compare the surface morphology of untreated and treated samples.

### 2.10. Mice and Management

Adult male C57BL/6 mice were used. All animals were weaned at 3 weeks of age and housed in open cages until 8 weeks old. They were then transferred to individually ventilated cages (IVCs). A total of 360 male mice were randomly assigned to ventilated cage racks in an environmentally controlled room. All mice were individually weighed and re-assigned using a completely randomized design. Each treatment had 6 replicates with 10 mice per pen. Animals were housed under a 14/10 h light/dark cycle (lights off at 20:00), temperature 22–24 °C, humidity 30–60%, and ventilation rate of 10 air changes per hour. They had ad libitum access to feed supplied by Maohua Biotechnology Co., Ltd.(Shenyang, China) and water. IVCs were provided with birch sawdust bedding and plastic shelters. Cages were changed every 7 days except for the initial 24 h post-delivery. All samples were collected between 14:00 and 18:00 (light phase). The experimental period was divided into two phases: the starter phase (days 0–28) and the finisher phase (days 29–56). The basal diet was formulated to meet the nutritional requirements of adult male mice except for VE. It consisted of corn, soybean meal, wheat flour, wheat bran, rice, chicken meal, yeast powder, soybean oil, dicalcium phosphate, limestone, salt, vitamins, amino acids, minerals, and trace elements. Treatment details are shown in Table 2.

### 2.11. Growth Performance Procedure

Animals and feed were weighed per pen on days 0, 28, and 56. Average body weight, feed intake, and feed-to-gain ratio were calculated for each phase. Average body weight gain (BWG) was calculated as the difference between the final live body weight and the initial live body weight. Mortalities and culled animals were excluded from BWG calculations. Average feed intake (FI) was calculated as the total feed consumption during the period minus the estimated feed consumed by animals that died. This estimation was based on the weight gain of the dead animal during its time in the trial and the estimated feed conversion ratio for that period. The average feed-to-gain ratio (F/G) was calculated as the total feed consumption divided by the weight gain of live animals and the gain of dead mice during that period.

### 2.12. Sampling

On day 56, six mice per group were euthanized. Blood samples were collected from the eyeball, and liver samples were collected. Serum was separated by centrifugation at 4 °C, 3000 *g* for 15 min, and stored at −20 °C. Livers were stored at −80 °C for subsequent analysis of aspartate aminotransferase, superoxide dismutase (SOD), total antioxidant capacity (TAOC), glutathione peroxidase (GSH-Px), hydrogen peroxide (H_2_O_2_), malondialdehyde (MDA), immunoglobulins G, M, A (IgG, IgM, IgA), tumor necrosis factor-α (TNF-α), interleukin-1β (IL-1β), interleukin-6 (IL-6), and related gene expression.

### 2.13. Assays

Commercial kits were used to determine the activities/levels of SOD, TAOC, GSH-Px, H_2_O_2_, MDA, IgG, IgM, IgA, TNF-α, IL-1β, and IL-6 in serum. All procedures were performed according to the manufacturers’ instructions.

### 2.14. Quantitative Real-Time PCR (qRT-PCR)

Relative qRT-PCR was used to detect the mRNA expression of SOD, CAT, GPX, *IL-1β*, TNF-α, and *IL-10* isolated from mouse liver tissue. Specificity was analyzed and confirmed based on dissociation curves. Primer sequences are listed in Table 3. Real-time PCR was performed using a Bio-Rad CFX96 Real-Time Detection System (Bio-Rad Laboratories, Inc., Hercules, CA, USA). The expression of target genes was normalized to the reference gene GAPDH. The conditions and protocol for real-time PCR were as described previously [20].

### 2.15. Statistics

All measurements were performed in triplicates. Origin 2022 software was used for graphing. Response surface data were modeled and analyzed by ANOVA using Design-Expert 13 software. Data were subjected to one-way ANOVA using the General Linear Model procedure of SAS software (SAS Institute, Cary, NC, USA, version 2008) to assess the main effects of treatments. Differences among means were separated using Duncan’s multiple range test at the *p* < 0.05 significance level.

## 3. Results

### 3.1. Major Components of Phenolic Substances in Grape Seeds

**Figure 1:**
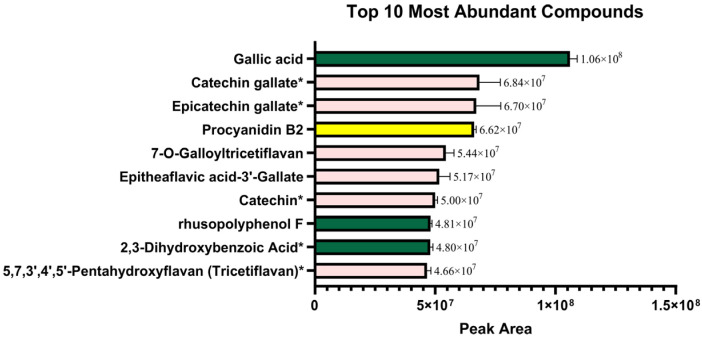
The top 10 polyphenolic compounds with highest contents in Kyoho grape seeds. Pink bars: flavanols; green bars: phenolic acids; yellow bars: proanthocyanidins. Asterisks (*) indicate compounds that are structural units or derivatives of PCs.

**Figure 2:**
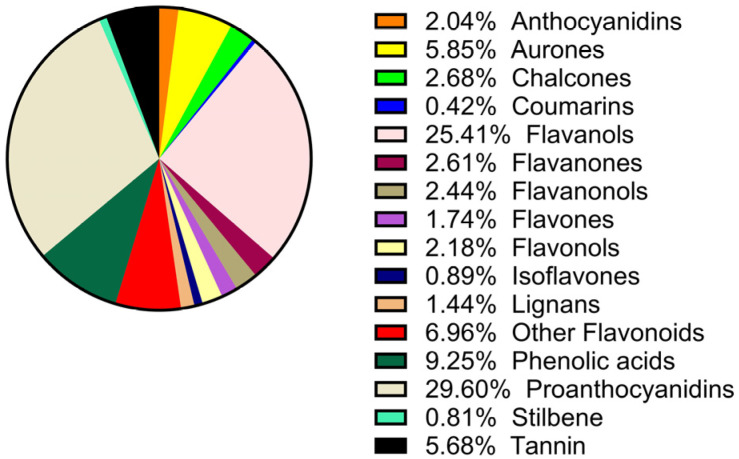
Secondary classification and relative abundance of polyphenolic compounds in Kyoho grape seeds.

As shown in Figure 1, the 10 most abundant compounds among polyphenols in Kyoho grape seeds were identified. Figure 2 further revealed that procyanidins accounted for 29.6% of the total phenolic substances based on secondary classification.

### 3.2. Single-Factor Studies

**Figure 3:**
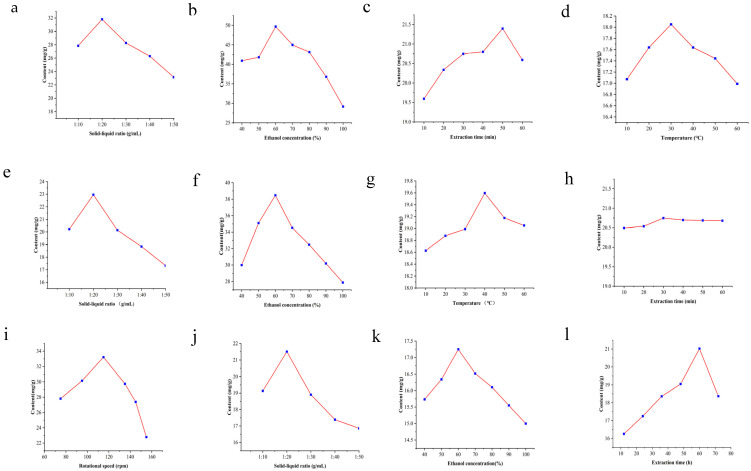
Analysis of the results of the one-factor experiment of the ultrasound-assisted, shaker-assisted and infiltration at low temperature. Note: Ultrasound-assisted extraction (**a**–**d**): Effects of (**a**) solid-to-liquid ratio (1:10–1:50 g/mL), (**b**) ethanol concentration (40–100%), (**c**) extraction time (10–60 min), and (**d**) temperature (10–60 °C) on PCs yield. Shaker-assisted extraction (**e**–**i**): Effects of (**e**) solid-to-liquid ratio (1:10–1:50 g/mL), (**f**) ethanol concentration (40–100%), (**g**) temperature (10–60 °C), (**h**) extraction time (10–60 min), and (**i**) rotational speed (75–155 rpm) on PCs yield. Low-temperature infiltration extraction (**j**–**l**): Effects of (**j**) solid-to-liquid ratio (1:10–1:50 g/mL), (**k**) ethanol concentration (40–100%), and (**l**) extraction time (12–72 h) on PCs yield. Data are presented as mean ± SD (n = 3).

As can be seen from Figure 3a, when extracting PCs by the ultrasound-assisted method, the extraction yield increased as the material–liquid ratio increased from 1:10 to 1:50 g/mL and then gradually decreased with the increase in material–liquid ratio. The PC content reached its maximum at the material–liquid ratio of 1:20 g/mL. Figure 3b shows that the highest content of PCs was found at an ethanol concentration of 60%, and when the ethanol concentration exceeded 60%, the PC content decreased as the ethanol concentration increased. Figure 3c and Figure 3d show that the optimal temperature and time for the extraction of PCs in univariate analysis were 30 °C and 50 min, respectively.

Figure 3e–i shows the extraction PCs by the shaker-assisted method. As shown in Figure 3e–i, it can be seen that the content of PCs exhibited a trend of increasing and then decreasing with increasing material–liquid ratio, ethanol concentration, temperature, time and rotational speed, but the change in PCs content over time was not significant.

Solid-to-liquid ratio, ethanol concentration, and time were the main factors affecting the low-temperature infiltration method. As shown in Figure 3j–l, the optimal extraction ranges for the low-temperature infiltration method of feed liquid ratio, ethanol concentration and time for extraction of PCs were 1:10 to 1:30 g/mL, 50% to 70% and 50 to 70 h, respectively.

### 3.3. Regression Modeling and Variance Analysis

Based on the Box–Behnken design principle, solid-to-liquid ratio, ethanol concentration, temperature, and time were determined as response factors for ultrasonic-assisted extraction. For shaker-assisted extraction, rotational speed was an additional factor. For low-temperature infiltration, only the solid-to-liquid ratio, ethanol concentration, and time were response factors. PC content was the response value. Response surface optimization experiments were conducted. Data were analyzed by ANOVA. The predicted R^2^ values for ultrasonic-assisted, shaker-assisted, and low-temperature infiltration methods were 0.8954, 0.9650, and 0.9978, respectively, which were in reasonable agreement with the adjusted R^2^ values (0.9585, 0.9301, and 0.9949), with differences less than 0.2. The coefficient of variation for the fit term was less than 5% for all three methods, indicating good model fit and reproducibility. Experimental designs and results are shown in Table A1, Table A2, Table A3, Table A4, Table A5 and Table A6.

Using Design-Expert V8.0.6 software, the experimental data from Table A1, Table A3 and Table A5 were linearly fitted, yielding the following regression equations for PCs content: Y1 = +35.98 − 1.49A − 3.95B + 0.1429C + 0.8005D + 0.8577AB − 0.2009AC − 1.48AD + 1.26BC + 0.0576BD − 3.94CD − 4.10A^2^ − 4.66B^2^ − 4.17C^2^ − 4.09D^2^, Y2 = +29.72 − 0.4312A − 1.40B + 1.18C − 1.43D + 1.48AB − 1.26AC − 2.37AD − 1.52BC + 1.50BD − 1.03CD − 5.18A^2^ − 4.02B^2^ − 5.04C^2^ − 4.05D^2^ and Y3 = +33.34 − 0.0716A − 2.89B + 1.41C + 0.4322AB − 0.2621AC + 0.4695BC − 4.68A^2^ − 4.20B^2^ − 4.84C^2^; Analysis of the quadratic regression equations is presented in Table A2, Table A4 and Table A6.

As can be seen from attachment Table A2, Table A4 and Table A6, the F-values of the model were 47.19 (*p* < 0.05), 27.6 (*p* < 0.05) and 345.53 (*p* < 0.05), and the F-values of the misfit terms were 1.93 (*p* > 0.05), 2.74 (*p* > 0.05) and 5.27 (*p* > 0.05), indicating that the model was well established. It can be used as the research result of PCs extraction process. As shown in attachment Table A2, the order of influence of factors on PC content in ultrasound-assisted extraction was B (material–liquid ratio) > A (ethanol concentration) > D (extraction time) > C (extraction temperature). Attachment Table A4 shows that the order of effect of factors on PC content in the shaker-oscillation-assisted method is B (material–liquid ratio) > C (temperature) > D (rotational speed) > A (ethanol concentration). Attachment Table A6 shows that the order of influence of factors on PC content in low-temperature infiltration extraction was B (material–liquid ratio) > C (time) > A (ethanol concentration).

### 3.4. Response Surface Analysis

**Figure 4:**
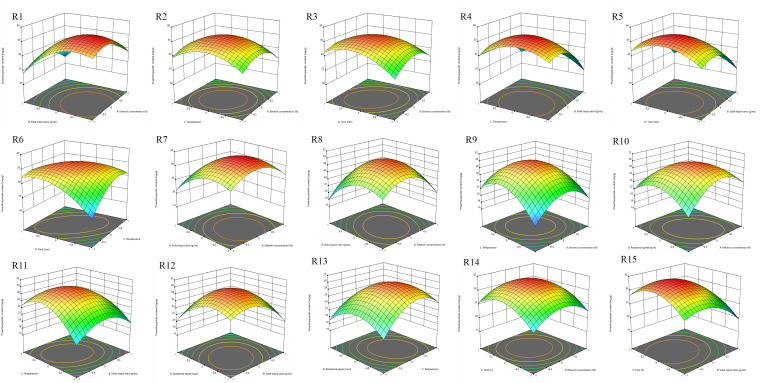
(**R1**–**R6**), the factors affecting PC yield during ultrasound-assisted extraction of PCs were investigated using response surface and contour plots: feed-to-liquid ratio versus ethanol concentration (**R1**), ethanol concentration versus extraction temperature (**R2**), ethanol concentration versus extraction time (**R3**), temperature versus feed-to-liquid ratio (**R4**), time versus feed-to-liquid ratio (**R5**), and extraction temperature versus extraction time (**R6**). (**R7**–**R12**), Response surface and contour plots were used to investigate the factors affecting PCs yield during shaker-assisted extraction of PCs: feed-to-liquid ratio versus ethanol concentration (**R7**), ethanol concentration versus extraction temperature (**R8**), ethanol concentration versus rotational speed (**R9**), temperature versus feed-to-liquid ratio (**R10**), rotational speed versus feed-to-liquid ratio (**R11**), and extraction temperature versus rotational speed (**R12**). (**R13**–**R15**), Response surface and contour plots were used to investigate the factors affecting the PC yield during PC extraction by low-temperature infiltration: feed-to-liquid ratio versus ethanol concentration (**R13**), ethanol concentration versus extraction time (**R14**), and time versus rotational speed (**R15**).

Response surface and contour plots illustrating the interactive effects of factors on PCs content for the three methods are shown in Figure 4. Results indicated significant interactions (*p* < 0.05) between ethanol concentration and time, and between solid-to-liquid ratio and temperature for the ultrasonic method. For the shaker method, significant interactions existed between all factors except temperature and rotational speed. For the low-temperature infiltration method, significant interactions were found between ethanol concentration and solid-to-liquid ratio, and between solid-to-liquid ratio and time.

### 3.5. Confirmation and Validation of Optimal Conditions

Using Design-Expert software, the model predicted the optimal conditions for ultrasonic-assisted extraction as: ethanol concentration 60%, solid-to-liquid ratio 1:20 g/mL, temperature 30 °C, time 50 min. For shaker-assisted extraction: ethanol 60%, solid-to-liquid ratio 1:20 g/mL, temperature 40 °C, rotational speed 115 rpm. For low-temperature infiltration: ethanol 60%, solid-to-liquid ratio 1:20 g/mL, time 60 h. Under these conditions, triplicate validation experiments were performed. For ultrasound-assisted extraction, the average PCs content was 33.77 ± 0.02 mg/g, which was in close agreement with the model-predicted value (35.97 mg/g). For shaker-assisted extraction, the average PC content was 30.19 ± 0.01 mg/g, compared with the predicted value (29.72 mg/g). For low-temperature infiltration, the average PC content was 33.76 ± 0.01 mg/g, compared with the predicted value (33.34 mg/g). The low coefficients of variation (<0.1%) for all three methods confirmed the reliability and reproducibility of the optimized extraction conditions.

### 3.6. Microstructural Analysis

**Figure 5:**
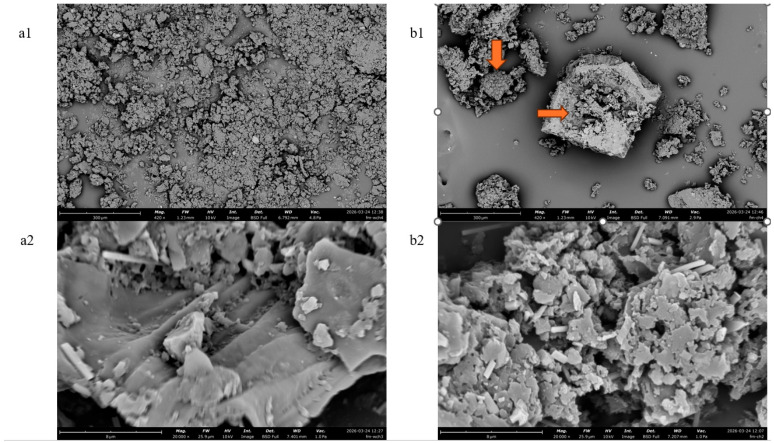
Scanning electron microscopy (SEM) images of grape seed powder before and after ultrasound-assisted extraction. (**a1**) Untreated sample (420×); (**a2**) untreated sample (20,000×); (**b1**) ultrasonically treated sample (420×); (**b2**) ultrasonically treated sample (20,000×). Arrows indicate surface cracks and pores induced by ultrasonication.

To investigate the physical effects of ultrasound-assisted extraction on grape seed cellular structure, SEM was employed to compare the morphology of untreated and ultrasonically treated grape seed powder. As shown in Figure 5(a1) (420×) and Figure 5(a2) (20,000×), untreated grape seed particles exhibited relatively smooth and intact surfaces with a compact structure. In contrast, after ultrasound-assisted extraction under the optimized conditions (60% ethanol, solid-to-liquid ratio 1:20 g/mL, 30 °C, 50 min), the treated samples (Figure 5(b1,b2)) displayed pronounced morphological alterations. The surfaces became rough and fragmented, exhibiting numerous cracks, pores, and dislodged cellular debris. These structural disruptions significantly increased the contact area between the solvent and the intracellular components, facilitating the release of proanthocyanidins from the seed matrix.

### 3.7. In Vitro Antioxidant Activity

**Figure 6:**
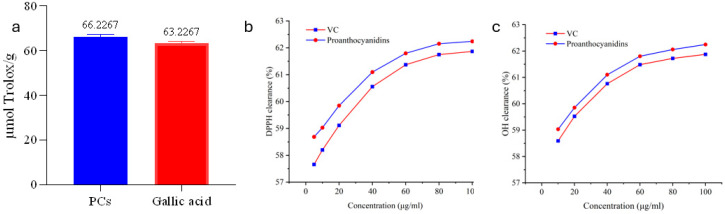
DPPH and •OH scavenging by PCs. Note: (**a**): DPPH and •OH scavenging activities of PCs expressed as μmol Trolox equivalents per gram of extract (μmol TE/g). VC and gallic acid served as positive controls. (**b**): Concentration-dependent DPPH radical scavenging activity of PCs and VC. (**c**): Concentration-dependent •OH scavenging activity of PCs and VC. Data are presented as mean ± SD (n = 3).

To enable quantitative comparison with widely used reference standards, the antioxidant activities were also expressed as μmol Trolox equivalents per gram of extract (μmol TE/g). As shown in Figure 6a, PCs exhibited higher DPPH scavenging activities compared to both Trolox and gallic acid.

As shown in Figure 6b, both PCs and VC exhibited concentration-dependent DPPH radical scavenging activity. Within the 5–100 μg/mL dose range, the scavenging activity increased progressively with concentration. However, within this dose range, the IC_50_ for PCs (4.54 μg/mL) was significantly lower than that for VC (IC_50_ = 7.02 μg/mL). Therefore, PCs exhibited stronger in vitro DPPH radical scavenging activity than VC.

The •OH scavenging results for different PC concentrations are shown in Figure 6c. The •OH scavenging capacity of PCs (IC_50_ = 5.47 μg/mL) was significantly higher than that of the positive control VC (IC_50_ = 9.21 μg/mL), indicating potent hydroxyl radical scavenging ability.

### 3.8. In Vivo Antioxidant Effects

The effects of dietary PCs and VE levels on mouse growth are shown in Table 4. Dietary treatments did not significantly affect the growth performance of mice fed diets containing PCs and VE.

The effects of dietary PCs and VE levels on serum and liver antioxidant indices in mice are shown in Table 5. Mice fed diets supplemented with PCs and VE showed significantly higher (*p* < 0.05) activities of SOD, TAOC, and GSH-Px in serum and liver compared to the control group, while H_2_O_2_ and MDA levels indicated the opposite trend.

**Figure 7:**
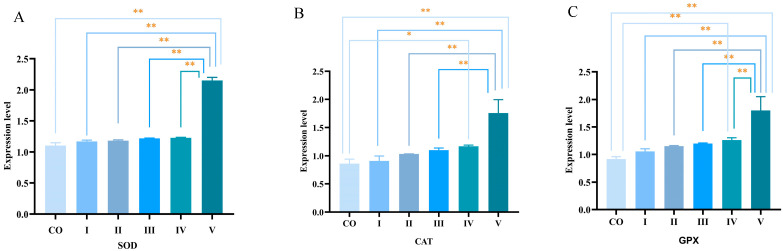
Effects of dietary PCs and VE supplementation on the mRNA expression levels of antioxidant enzymes in mouse liver. (**A**) SOD, (**B**) CAT, and (**C**) GPX mRNA expression were determined by qRT-PCR and normalized to the housekeeping gene GAPDH. (* *p* < 0.05, ** *p* < 0.01).

As shown in Figure 7, mice fed the diet containing 100 mg/kg PCs exhibited significantly higher hepatic mRNA expression levels of SOD, CAT, and GPX compared to other groups (*p* < 0.05).

### 3.9. Immunomodulatory Effects on Mice

The effects of dietary PCs and VE levels on immune organ indices in mice are shown in Table 6. No significant changes were observed in the indices of various immune organs (liver, kidney, spleen, pancreas) in mice fed diets containing PCs and VE.

The effects of dietary PCs and VE levels on immune parameters in mouse serum and liver are presented in Table 7. Compared with the control group, mice fed the diet containing 100 mg/kg PCs exhibited significantly lower levels of IgG, IgM, IgA, TNF-α, IL-1β, and IL-6 in serum and liver (*p* < 0.05).

**Figure 8:**
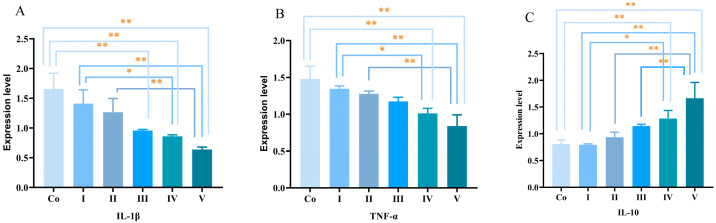
Effects of dietary PCs and VE supplementation on the mRNA expression levels of inflammatory cytokines in mouse liver. (**A**) *IL-1β*, (**B**) *TNF-α*, and (**C**) *IL-10* mRNA expression were determined by qRT-PCR and normalized to GAPDH. * *p* < 0.05, ** *p* < 0.01.

As shown in Figure 8, mice fed the diet containing 100 mg/kg PCs exhibited significantly lower hepatic mRNA expression levels of *IL-1β* and *TNF-α* compared to other groups (*p* < 0.05), whereas *IL-10* expression showed the opposite trend.

## 4. Discussion

### 4.1. Response Surface

Water/ethanol mixtures have been employed for the extraction of PCs from grape stalks and grape seed meal [21], grape seeds [22,23], grape skins and stalks [24], and red grape pomace [25]. The varying trends and optimal ethanol levels observed in each trial’s recorded impact of different factors on PCs extraction yield indicate that the optimal conditions for extracting PCs from different species should be determined on a case-by-case basis, with no universal model describing the ideal parameters. This experiment used Kyoho grape seeds as raw material and compared the effects of UAE, SAE, and LTI on PC yield.

Optimization revealed that among the three extraction methods, while holding other parameters constant (solid-to-liquid ratio 1:30 g/mL, temperature 30 °C, extraction time 30 min), the optimal ethanol concentration for extracting PCs was 60%. Beyond 60%, the extracted PCs content decreased. This may be because increased ethanol concentration disrupts interactions such as hydrogen bonding and hydrophobic non-covalent bonds, leading to PC dissolution [26]. Some studies also report optimal ethanol concentrations for PCs extraction from grape stalks ranging from 44.2% to 53.1% [27]. Compared to our results, this slightly lower optimal concentration may be attributed to differences in the source material.

To optimize the PC extraction capacity from grape seeds, each time range was assessed with other parameters fixed (solid-to-liquid ratio 1:30 g/mL, ethanol concentration 60%, temperature 30 °C). In LTI, PC content increased with time, reaching a peak at 60 h, after which it decreased with further time (Figure 3l). This could be due to ethanol evaporation over prolonged periods. The effect of extraction time on PCs was not significant in SAE. In contrast, PC content significantly increased with 50 min of ultrasonic treatment, then gradually decreased with further time. Patil [28] used ultrasound-assisted extraction to enhance camptothecin yield from Nothapodytes nimmoniana. However, prolonged ultrasonication may lead to polyphenol degradation, thereby reducing the extraction yield [29]. Therefore, 50 min was selected as the optimal extraction time for UAE.

With other parameters fixed (solid-to-liquid ratio 1:30 g/mL, ethanol concentration 60%, extraction time 30 min), the effect of extraction temperature on PC yield was optimized. For both UAE and SAE, PC content initially increased and then decreased with rising temperature (Figure 3d,j), indicating that high temperatures are unfavorable for PC extraction. This is likely because the hydroxyl groups in phenolic compounds are prone to oxidation and condensation reactions at elevated temperatures [30]. Consequently, the temperature range selected for the UAE was 20–40 °C, and for SAE was 30–50 °C.

As revealed by SEM images, although UAE enhanced PC yield by disrupting the surface structure of grape seeds, excessive ultrasonication time posed a risk of polyphenol degradation [31]. Ultrasonic cavitation generates localized high temperatures and pressures, as well as the formation of ROS, which can potentially oxidize phenolic compounds [29]. In the present study, PC yield increased with extraction time up to 50 min but decreased thereafter (Figure 3d), suggesting that excessive ultrasonication leads to degradation [32]. This decline is likely attributable to thermal effects, as the extraction temperature (30 °C) was relatively moderate, making ROS-mediated oxidation a more plausible cause. Similar observations have been reported for the ultrasound-assisted extraction of polyphenols from various plant matrices, where optimal extraction times typically range from 30 to 60 min [33,34]. Therefore, careful optimization of extraction time is critical to balance yield enhancement against degradation.

This experiment employed RSM to optimize the conditions for UAE, SAE, and LTI for extracting PCs from Kyoho grape seeds, achieving high PC yields. UAE proved more effective than SAE and LTI. Under the optimal UAE conditions (ultrasonic time 50 min, temperature 30 °C, ethanol concentration 60%, solid-to-liquid ratio 1:20 g/mL), the actual PCs yield was 35.97 mg/g, which closely matched the model prediction. Statistical analysis with a high correlation coefficient confirmed the validity and reproducibility of the model.

### 4.2. Antioxidant Activity of PCs

Oxidative stress is a disturbance caused by an overproduction of reactive oxidative species (e.g., free radicals) exceeding the cell’s intrinsic antioxidant capacity. It can lead to various adverse outcomes such as cellular damage, inflammation, mitochondrial impairment, and neuronal injury [35]. PCs from grape seeds are flavonoid polyphenolic compounds that act as potent free radical scavengers against various radicals (e.g., DPPH, ABTS, and •OH) [36]. In studies on the in vitro antioxidant activity of grape seed polyphenols, PCs were found to have a lower IC_50_ value for DPPH scavenging than the positive control VE [37]. The results of this experiment show that within the 5–100 μg/mL dose range, the IC_50_ values of PCs for scavenging both DPPH and •OH were significantly lower than those of VC. Furthermore, PCs demonstrated superior radical scavenging capacity compared to both Trolox and gallic acid. Other studies also suggest that PCs, abundant in grape seeds, exhibit stronger free radical scavenging capacity than VE and VC [38], with their antioxidant capacity reported to be 20 times that of VE and 50 times that of VC [39].

The superior antioxidant activity of PCs compared to VE and VC observed in this study can be attributed to their unique chemical structure. Proanthocyanidins are oligomeric and polymeric flavan-3-ols, predominantly composed of catechin and epicatechin units. The presence of multiple phenolic hydroxyl (-OH) groups in their molecular structure allows for efficient hydrogen atom donation to neutralize free radicals [36,40]. Upon donating a hydrogen atom, the resulting phenoxyl radical can be stabilized through resonance and intramolecular hydrogen bonding, preventing further radical propagation. In contrast, VE contains only a single phenolic hydroxyl group, and VC, while possessing an enediol structure, lacks the polymeric structure that enables the “radical sink” effect characteristic of proanthocyanidins [41]. Furthermore, the ortho-dihydroxy (catechol) or ortho-trihydroxy (galloyl) groups present on the B-ring of PCs can chelate metal ions, thereby inhibiting Fenton reaction-induced hydroxyl radical formation—an additional antioxidant mechanism not shared by VE or VC [42]. From these results, it can be preliminarily inferred that PCs could serve as potent antioxidants in feed additives to mitigate oxidative stress.

This experiment not only confirmed the superior in vitro radical scavenging capacity of PCs extracted from Kyoho grape seeds but also found through in vivo studies that dietary PCs supplementation enhanced the antioxidant status of mice without affecting their growth performance. It has been reported that grape seed PC extract protects mouse kidneys from cadmium- and cisplatin-induced oxidative damage [43,44]. Proanthocyanidins can reverse the reduction in liver peroxidase, superoxide dismutase, and erythrocyte glutathione peroxidase activities caused by Plasmodium berghei NK65 infection, thereby enhancing antioxidant capacity in erythrocytes and livers of infected mice [45].The underlying mechanism likely involves integrated regulation of cellular redox balance, particularly through activating key defensive transcription factors such as nuclear factor erythroid 2-related factor 2 (Nrf2) [46]. In a hypoxia-reoxygenation model in renal tubular epithelial cells, it was demonstrated that GSPE significantly upregulated Nrf2 nuclear translocation and HO-1 expression, concomitant with reduced oxidative stress [47]. Under basal conditions, Nrf2 is sequestered in the cytoplasm by Keap1. Upon exposure to PCs, Nrf2 dissociates from Keap1, translocates to the nucleus, and binds to the antioxidant response element (ARE), initiating transcription of downstream target genes [48]. Activation of the Nrf2 pathway is a well-recognized mechanism by which PCs enhance the endogenous antioxidant defense capacity of the body. Moreover, Nrf2 is a key transcription factor that regulates the expression of antioxidant enzymes such as SOD, CAT, and GPx [49,50]. In the present study, the significant upregulation of SOD, CAT, and GPX mRNA expression in the livers of mice fed PCs-supplemented diets (Figure 7) is consistent with Nrf2 pathway activation, suggesting that PCs may exert their antioxidant effects through this transcriptional mechanism.

### 4.3. Immunomodulatory Effects of PCs

Grape seed PCs are natural compounds with diverse biological activities, including anti-inflammatory, antioxidant, and anti-tumor properties [51]. Research indicates that grape seed PCs significantly reduce IL-8 levels in gastric epithelial cells infected with Helicobacter pylori [52]. In the present experiment, dietary supplementation with varying levels of PCs and VE did not significantly alter immune organ indices in mice. However, it significantly affected the levels of immunoglobulins (IgM, IgG, IgA) and inflammatory cytokines (TNF-α, IL-1β, IL-6) in serum and liver, upregulating the *IL-10* gene while downregulating *IL-1β* and *TNF-α* genes. A transcriptomic analysis comparing rats fed a high-fat diet (HFD) during pregnancy and lactation with or without grape seed PCs supplementation (HFD-GSP vs. HFD) identified 238 differentially expressed genes, most of which were related to immune function and inflammatory response [53]. It is reported that grape seed PCs exert anti-inflammatory effects in toxin-stimulated RAW 264.7 macrophages by inhibiting the NF-κB signaling pathway [54]. Inhibition of the NF-κB pathway is a key mechanism underlying the anti-inflammatory effects of PCs. NF-κB is a central regulator of inflammatory responses, and its activation leads to the transcription of pro-inflammatory cytokines such as TNF-α, IL-1β, and IL-6 [42]. Reactive oxygen species (ROS) act as second messengers that can activate NF-κB, establishing a link between oxidative stress and inflammation [55]. Wang et al. [56]. demonstrated that GSPE can alleviate recurrent colitis by inhibiting the NF-κB signaling pathway, reducing the nuclear translocation of NF-κB p65, and suppressing the production of TNF-α, IL-1β and ROS [41,57]. In the current study, the reduced serum and liver levels of TNF-α, IL-1β, and IL-6 (Table 7), together with the downregulation of *IL-1β* and *TNF-α* gene expression (Figure 8), strongly suggest that PCs inhibit the NF-κB signaling pathway. Based on these results, it is hypothesized that PCs may modulate immune function, at least in part, by activating the Nrf2 signaling pathway, thereby suppressing oxidative stress in vivo. Integrating the previously described antioxidant effects of PCs, we further postulate that by regulating in vivo oxidative stress through antioxidant and anti-inflammatory actions, PCs have the potential to replace VE as an effective antioxidant in feed additives.

Crosstalk between Nrf2 and NF-κB pathways provides a mechanistic link between the antioxidant and anti-inflammatory effects of PCs [58]. Studies have shown that Nrf2 interferes with the transcriptional activity of NF-κB through multiple mechanisms, including competing for transcriptional coactivators and inhibiting ROS-mediated NF-κB activation. Blocking the NF-κB signaling pathway not only enhances anti-inflammatory activity, but also activates the MAPKs and Nrf2/HO-1 signaling pathways to improve antioxidant capacity, inhibit the nuclear translocation of NF-κB, and increase the nuclear translocation of Nrf2 [57,59]. Therefore, the enhanced antioxidant capacity resulting from Nrf2 activation may indirectly attenuate NF-κB-driven inflammation. This integrated regulation likely contributes to the superior efficacy of PCs compared to VE, as observed in this study.

In summary, while direct protein-level confirmation is not provided, the combination of our gene expression data, biochemical indicators, and robust literature evidence strongly supports a model in which PCs exert their antioxidant and immunomodulatory effects through activation of the Nrf2 pathway and inhibition of the NF-κB pathway. Future studies employing techniques such as Western blotting and immunofluorescence will be valuable to further validate this mechanistic framework.

## 5. Conclusions

This study demonstrated that procyanidins are the predominant phenolic components in Kyoho grape seeds, accounting for 29.6% of the total polyphenolic content. Ultrasonic-assisted extraction under optimized conditions (60% ethanol, solid-to-liquid ratio 1:20 g/mL, 30 °C, 50 min) proved superior to both shaker-assisted and low-temperature infiltration methods. The extracted PCs exhibited potent antioxidant activity in vitro, surpassing VC and Trolox standards. In vivo, dietary PCs supplementation enhanced antioxidant enzyme activities (SOD, GSH-Px, TAOC) and upregulated SOD, CAT, and GPX gene expression while reducing pro-inflammatory cytokines (TNF-α, IL-1β, IL-6) and modulating *IL-1β*, *TNF-α*, and *IL-10* expression in mice. These findings suggest that PCs exert their effects through coordinated activation of the Nrf2 antioxidant pathway and inhibition of the NF-κB inflammatory pathway. Given their superior efficacy compared to VE and VC, combined with low toxicity and cost-effectiveness, PCs derived from grape seeds show promise as a natural feed additive for enhancing antioxidant capacity and immune function in animals.

## Figures and Tables

**Table 1:** Factors and levels for the Response Surface Experimental Design of ultrasonic-assisted, shaker-assisted, and low-temperature infiltration methods.

	Coded Levels	Uncoded Levels				
		Ethanol Concentration (%)	Solid–Liquid Ratio (g/mL)	Temperature (°C)	Time (min)	Rotational Speed (rpm)
shaker-assisted methods	−1	50	1:10	20	40	
	0	60	1:20	30	50	
	1	70	1:30	40	60	
shaker-assisted	−1	50	1:10	30		95
	0	60	1:20	40		115
	1	70	1:30	50		135
low-temperature maceration	−1	50	1:10		48 ^1^	
	0	60	1:20		60 ^1^	
	1	70	1:30		72 ^1^	

Note: Superscript 1 in the table represents the unit h.

**Table 2:** Experimental design.

Treatment	Details
Control	Negative Control (NC)
I	NC + VE (100 mg/kg)
II	NC + VE (75 mg/kg) + GSE (25 mg/kg)
III	NC + VE (50 mg/kg) + GSE (50 mg/kg)
IV	NC + VE (25 mg/kg) + GSE (75 mg/kg)
V	NC + GSE (100 mg/kg)

**Table 3:** Primer sequence information.

Primer Name	Nucleotide Sequences (5′→3′)	GenBank Accession No.
*SOD(F)*	GAACCATCCACTTCGAGCAG	NM_000454.5
*SOD(R)*	CAACATGCCTCTCTTCATCCG	
*CAT(F)*	ATAGCCAGAAGAGAAACCCACA	NM_009804.2
*CAT(R)*	GCCTCTCCATCTGCATTAACCAA	
*GPX(F)*	ACAGTCCACCGTGTATGCC	NM_001329860.1
*GPX(R)*	AGCACCACCAGTCCACGAG	
*GAPDH(F)*	GCCTCCTCCAATTCAACCCTT	NM_001289726.2
*GAPDH(R)*	AACAATCTCCACTTTGCCACT	
*IL-1β(F)*	TTCAGGCAGGCAGTATCACTCATTG	NM_008361.4
*IL-1β(R)*	TGTCGTTGCTTGGTTCTCCTTGTAC	
*TNF-α(F)*	GGACTAGCCAGGAGGGAGAACAG	NM_001278601.1
*TNF-α(R)*	GCCACAAGCAGGAATGAGAAGAGG	
*IL-10(F)*	CTGCTATGCTGCCTGCTCTTACTG	NM_010548.2
*IL-10(R)*	AGCCGCATCCTGAGGGTCTTC	

**Table 4:** Effects of dietary PCs and VE levels on the growth performance of mice.

Items	Groups					
	Control	I	II	III	IV	V
Average initial weight (g)	41.46 ± 0.62	42.47 ± 0.53	42.03 ± 0.83	43.00 ± 0.85	43.04 ± 0.35	41.65 ± 1.17
Pre-production (1–28 d)						
Average daily feed intake (d/g)	6.88 ± 0.11	7.06 ± 0.18	7.00 ± 0.04	6.98 ± 0.06	7.01 ± 0.06	6.83 ± 0.14
Average daily weight gain (d/g)	0.25 ± 0.02	0.24 ± 0.01	0.25 ± 0.01	0.21 ± 0.02	0.25 ± 0.02	0.23 ± 0.02
Feed conversion ratio	25.71 ± 1.79	29.06 ± 2.49	28.28 ± 1.42	31.57 ± 1.96	28.08 ± 2.44	30.63 ± 2.75
Later period (29–56 d)						
Average daily feed intake (d/g)	6.51 ± 0.16	6.24 ± 0.06	6.60 ± 0.18	6.52 ± 0.21	6.23 ± 0.08	6.66 ± 0.15
Average daily weight gain (d/g)	0.10 ± 0.02	0.11 ± 0.01	0.13 ± 0.01	0.14 ± 0.01	0.11 ± 0.01	0.13 ± 0.01
Feed conversion ratio	54.26 ± 4.79	51.51 ± 8.03	54.02 ± 4.73	55.87 ± 1.64	53.63 ± 2.80	54.44 ± 1.74

**Table 5:** Effects of dietary PCs and VE levels on serum and liver antioxidant indices in mice.

Items	Groups					
	Control	I	II	III	IV	V
Serum						
SOD (U/mL)	35.04 ± 0.85 ^e^	47.19 ± 0.63 ^d^	51.44 ± 3.49 ^cd^	54.08 ± 1.25 ^c^	60.08 ± 1.35 ^b^	79.95 ± 1.93 ^a^
TAOC (U/mL)	8.62 ± 0.144 ^d^	9.23 ± 0.19 ^cd^	9.61 ± 0.26 ^c^	10.50 ± 0.16 ^b^	10.90 ± 0.23 ^ab^	11.42 ± 0.39 ^a^
GSH-PX (U/mL)	250.53 ± 13.25 ^d^	325.25 ± 8.91 ^c^	326.24 ± 16.26 ^c^	353.95 ± 11.12 ^c^	408.58 ± 10.87 ^b^	479.48 ± 18.98 ^a^
H_2_O (U/mL)	59.19 ± 2.88 ^a^	53.51 ± 1.28 ^b^	48.69 ± 2.28 ^b^	42.82 ± 2.57 ^c^	36.52 ± 0.67 ^d^	32.85 ± 0.88 ^d^
MDA (nmol/mL)	5.09 ± 0.21 ^a^	4.76 ± 0.09 ^ab^	4.50 ± 0.08 ^bc^	4.23 ± 0.09 ^c^	3.66 ± 0.18 ^d^	3.38 ± 0.06 ^d^
Liver						
SOD (U/mL)	9.42 ± 0.24 ^c^	11.99 ± 0.41 ^b^	12.43 ± 0.32 ^b^	12.58 ± 0.79 ^b^	13.20 ± 0.54 ^a^	15.63 ± 0.76 ^a^
TAOC (U/mL)	5.86 ± 0.25 ^d^	6.68 ± 0.37 ^cd^	6.93 ± 0.21 ^bc^	7.72 ± 0.20 ^ab^	7.91 ± 0.24 ^a^	8.47 ± 0.51 ^a^
GSH-PX (U/mL)	62.96 ± 5.73 ^b^	82.85 ± 4.98 ^a^	84.78 ± 3.81 ^a^	89.54 ± 3.42 ^a^	90.84 ± 4.54 ^a^	95.92 ± 4.81 ^a^
H_2_O (U/mL)	27.70 ± 2.46 ^a^	24.10 ± 1.44 ^b^	21.53 ± 1.06 ^b^	17.36 ± 0.29 ^c^	16.01 ± 0.73 ^c^	13.48 ± 0.95 ^c^
MDA (nmol/mL)	2.91 ± 0.32 ^a^	1.96 ± 0.20 ^b^	1.14 ± 0.14 ^c^	0.99 ± 0.11 ^c^	0.91 ± 0.07 ^c^	0.85 ± 0.01 ^c^

Note: In the same row, different small letters mean notable difference (*p* < 0.05), the same or no letters indicate no significant difference (*p* > 0.05).

**Table 6:** Effects of dietary PCs and VE levels on immune organ indices in mice.

Items	Groups					
	Control	I	II	III	IV	V
liver	4.94 ± 0.12	4.98 ± 0.09	5.02 ± 0.09	5.18 ± 0.14	5.14 ± 0.12	4.99 ± 0.11
kidney	1.53 ± 0.03	1.52 ± 0.03	1.51 ± 0.03	1.58 ± 0.05	1.52 ± 0.03	1.54 ± 0.04
spleen	0.44 ± 0.05	0.36 ± 0.04	0.37 ± 0.04	0.37 ± 0.04	0.44 ± 0.05	0.41 ± 0.04
pancreas	0.48 ± 0.03	0.50 ± 0.03	0.50 ± 0.03	0.52 ± 0.02	0.48 ± 0.03	0.46 ± 0.03

**Table 7:** Effects of dietary PCs and VE levels on serum and liver immune parameters in mice.

Items	Groups					
	Control	I	II	III	IV	V
Serum						
IgG (g/L)	3.07 ± 0.05 ^a^	2.82 ± 0.25 ^ab^	2.69 ± 0.28 ^ab^	2.61 ± 0.15 ^ab^	2.58 ± 0.11 ^ab^	2.40 ± 0.11 ^b^
IgM (g/L)	0.44 ± 0.01 ^a^	0.39 ± 0.01 ^a^	0.35 ± 0.01 ^ab^	0.32 ± 0.01 ^ab^	0.31 ± 0.01 ^b^	0.27 ± 0.01 ^c^
IgA (g/L)	1.74 ± 0.02 ^a^	1.56 ± 0.03 ^ab^	1.54 ± 0.03 ^ab^	1.51 ± 0.02 ^ab^	1.38 ± 0.02 ^b^	1.27 ± 0.01 ^c^
TNFα (pg/mL)	67.22 ± 2.67 ^a^	58.44 ± 2.18 ^a^	57.42 ± 1.96 ^a^	53.91 ± 1.91 ^ab^	51.31 ± 2.19 ^ab^	44.00 ± 1.34 ^b^
IL-1β (pg/mL)	26.74 ± 1.52 ^a^	23.79 ± 1.54 ^ab^	23.11 ± 0.82 ^ab^	18.77 ± 0.75 ^ab^	14.27 ± 1.27 ^b^	11.93 ± 0.76 ^c^
IL-6 (pg/mL)	155.30 ± 4.26 ^a^	148.62 ± 3.24 ^a^	136.91 ± 1.37 ^a^	129.71 ± 1.38 ^a^	120.91 ± 2.87 ^a^	111.11 ± 3.12 ^b^
Liver						
IgG (g/L)	1.16 ± 0.06 ^a^	1.13 ± 0.02 ^a^	1.11 ± 0.12 ^a^	1.10 ± 0.10 ^a^	0.86 ± 0.08 ^b^	0.84 ± 0.08 ^b^
IgM (g/L)	0.17 ± 0.01 ^a^	0.16 ± 0.01 ^a^	0.14 ± 0.01 ^b^	0.13 ± 0.01 ^b^	0.12 ± 0.01 ^b^	0.11 ± 0.01 ^b^
IgA (g/L)	0.52 ± 0.05 ^a^	0.45 ± 0.02 ^a^	0.44 ± 0.04 ^a^	0.43 ± 0.03 ^a^	0.42 ± 0.04 ^a^	0.28 ± 0.02 ^b^
TNFα (pg/mL)	13.66 ± 0.61 ^a^	9.49 ± 0.69 ^b^	9.18 ± 0.71 ^b^	8.49 ± 0.95 ^b^	8.00 ± 0.82 ^b^	7.94 ± 0.81 ^b^
IL-1β (pg/mL)	7.78 ± 0.13 ^a^	6.10 ± 0.72 ^b^	5.92 ± 0.54 ^b^	5.70 ± 0.68 ^b^	5.48 ± 0.57 ^b^	4.45 ± 0.18 ^b^
IL-6 (pg/mL)	67.51 ± 2.04 ^a^	57.81 ± 3.93 ^b^	57.44 ± 3.43 ^b^	54.61 ± 4.05 ^b^	50.57 ± 3.77 ^b^	47.49 ± 1.75 ^b^

Note: In the same row, different small letters mean notable difference (*p* < 0.05), the same or no letters indicate no significant difference (*p* > 0.05).

## Data Availability

The original contributions presented in this study are included in the article. Further inquiries can be directed to the corresponding author.

## Abbreviations

The following abbreviations are used in this manuscript:

PCs	proanthocyanidins
RSM	response surface methodology
DPPH	2,2-diphenyl-1-picrylhydrazyl
•OH	hydroxyl
VE	vitamin E
VC	vitamin C
SOD	superoxide dismutase
GSH-PX	glutathione peroxidase
*IL-10*	inflammation factor 10
IgG	immunoglobulin G
IgA	immunoglobulin A
IgM	immunoglobulin M
*TNF-α*	tumor necrosis factor α
*IL-1β*	inflammation factor 1β

## Appendix A

**Table A1:** Design and results of response surface experiments for ultrasound-assisted method.

Std	Ethanol Concentration (%)	Solid–Liquid Ratio (g/mL)	Temperature (°C)	Time (min)	PCs Content (mg/g)
5	0	0	−1	−1	21.68
12	1	0	0	1	25.98
29	0	0	0	0	35.41
2	1	−1	0	0	28.41
13	0	−1	−1	0	32.53
21	0	−1	0	−1	30.93
22	0	1	0	−1	21.81
24	0	1	0	1	23.99
7	0	0	−1	1	31.21
18	1	0	−1	0	26.15
17	−1	0	−1	0	30.17
6	0	0	1	−1	31.27
15	0	−1	1	0	29.72
20	1	0	1	0	25.19
11	−1	0	0	1	31.16
16	0	1	1	0	24.76
27	0	0	0	0	36.50
14	0	1	−1	0	22.54
3	−1	1	0	0	23.49
25	0	0	0	0	36.77
1	−1	−1	0	0	32.42
26	0	0	0	0	35.01
9	−1	0	0	−1	27.10
4	1	1	0	0	22.91
19	−1	0	1	0	30.02
8	0	0	1	1	25.03
28	0	0	0	0	36.16
23	0	−1	0	1	32.87
10	1	0	0	−1	27.85

**Table A2:** Significance test and analysis of variance of regression models for ultrasound-assisted methods.

Source	Sum of Squares	df	Mean Square	F-Value	*p*-Value	
Model	603.22	14	43.09	47.19	<0.0001	significant
A-Ethanol concentration	26.57	1	26.57	29.10	<0.0001	
B-Solid–liquid ratio	187.13	1	187.13	204.96	<0.0001	
C-Temperature	0.2452	1	0.2452	0.2686	0.6124	
D-Time	7.69	1	7.69	8.42	0.0116	
AB	2.94	1	2.94	3.22	0.0942	
AC	0.1614	1	0.1614	0.1768	0.6805	
AD	8.80	1	8.80	9.64	0.0078	
BC	6.31	1	6.31	6.91	0.0198	
BD	0.0133	1	0.0133	0.0146	0.9057	
CD	62.17	1	62.17	68.10	<0.0001	
A^2^	108.82	1	108.82	119.19	<0.0001	
B^2^	140.56	1	140.56	153.95	<0.0001	
C^2^	112.68	1	112.68	123.42	<0.0001	
D^2^	108.74	1	108.74	119.10	<0.0001	
Residual	12.78	14	0.9130			
Lack of Fit	10.59	10	1.06	1.93	0.2746	not significant
Pure Error	2.19	4	0.5476			
Cor Total	616.00	28	43.09			

**Table A3:** Response surface experimental design and results of the shaker-assisted method.

Std	Ethanol Concentration (%)	Solid–Liquid Ratio (g/mL)	Temperature (°C)	Rotational Speed (rpm)	Proanthocyanidin Content (mg/g)
29	0	0	0	0	29.59
5	0	0	−1	−1	18.91
18	1	0	−1	0	18.72
17	−1	0	−1	0	17.40
20	1	0	1	0	19.45
4	1	1	0	0	18.98
3	−1	1	0	0	17.91
16	0	1	1	0	19.54
1	−1	−1	0	0	24.36
24	0	1	0	1	19.19
14	0	1	−1	0	21.25
28	0	0	0	0	28.73
8	0	0	1	1	19.62
27	0	0	0	0	29.39
12	1	0	0	1	17.23
9	−1	0	0	−1	19.31
15	0	−1	1	0	23.39
21	0	−1	0	−1	27.46
22	0	1	0	−1	20.41
13	0	−1	−1	0	19.04
10	1	0	0	−1	24.53
19	−1	0	1	0	23.15
7	0	0	−1	1	19.19
6	0	0	1	−1	23.48
25	0	0	0	0	30.64
11	−1	0	0	1	21.48
26	0	0	0	0	30.25
2	1	−1	0	0	19.52
23	0	−1	0	1	20.25

**Table A4:** Significance test and analysis of variance of regression model of shaker-assisted method.

Source	Sum of Squares	df	Mean Square	F-Value	*p*-Value	
Model	482.05	14	34.43	27.60	<0.0001	significant
A-Ethanol concentration	2.23	1	2.23	1.79	0.2024	
B-Solid–liquid ratio	23.36	1	23.36	18.72	0.0007	
C-Temperature	16.64	1	16.64	13.33	0.0026	
D-Rotational speed	24.51	1	24.51	19.64	0.0006	
AB	8.77	1	8.77	7.03	0.0190	
AC	6.32	1	6.32	5.07	0.0410	
AD	22.39	1	22.39	17.95	0.0008	
BC	9.19	1	9.19	7.36	0.0168	
BD	8.98	1	8.98	7.20	0.0178	
CD	4.28	1	4.28	3.43	0.0853	
A^2^	173.85	1	173.85	139.34	<0.0001	
B^2^	104.79	1	104.79	83.99	<0.0001	
C^2^	164.90	1	164.90	132.17	<0.0001	
D^2^	106.50	1	106.50	85.36	<0.0001	
Residual	17.47	14	1.25			
Lack of Fit	15.24	10	1.52	2.74	0.1716	not significant
Pure Error	2.22	4	0.5558			
Cor Total	499.52	28				

**Table A5:** Design and results of low-temperature infiltration response surface experiments.

Std	Ethanol Concentration (%)	Solid–Liquid Ratio(g/mL)	Time (min)	Proanthocyanidin Content (mg/g)
13	0	0	0	33.34
8	1	0	1	24.94
6	1	0	−1	22.61
2	1	−1	0	27.11
17	0	0	0	33.16
4	1	1	0	21.61
16	0	0	0	33.24
3	−1	1	0	20.93
7	−1	0	1	25.56
14	0	0	0	33.69
11	0	−1	1	27.82
5	−1	0	−1	22.17
1	−1	−1	0	28.16
10	0	1	−1	19.83
12	0	1	1	23.56
9	0	−1	−1	25.98
15	0	0	0	33.25

**Table A6:** Significance test and analysis of variance of regression model of low temperature infiltration method.

Source	Sum of Squares	df	Mean Square	F-Value	*p*-Value	
Model	381.18	9	42.35	345.53	<0.0001	significant
A-Ethanol concentration	0.0410	1	0.0410	0.3346	0.5811	
B-Solid–liquid ratio	66.93	1	66.93	546.07	<0.0001	
C-Time	15.98	1	15.98	130.36	<0.0001	
AB	0.7473	1	0.7473	6.10	0.0429	
AC	0.2747	1	0.2747	2.24	0.1781	
BC	0.8816	1	0.8816	7.19	0.0315	
A^2^	92.38	1	92.38	753.65	<0.0001	
B^2^	74.42	1	74.42	607.17	<0.0001	
C^2^	98.46	1	98.46	803.31	<0.0001	
Residual	0.8580	7	0.1226			
Lack of Fit	0.6847	3	0.2282	5.27	0.0711	not significant
Pure Error	0.1733	4	0.0433			
Cor Total	382.03	16				
